# Real-Time External Respiratory Motion Measuring Technique Using an RGB-D Camera and Principal Component Analysis †

**DOI:** 10.3390/s17081840

**Published:** 2017-08-09

**Authors:** Udaya Wijenayake, Soon-Yong Park

**Affiliations:** School of Computer Science and Engineering, Kyungpook National University, 80 Daehakro, Bukgu, Daegu 41566, Korea; udaya@vision.knu.ac.kr

**Keywords:** respiratory motion, radiotherapy, RGB-D camera, principal component analysis (PCA)

## Abstract

Accurate tracking and modeling of internal and external respiratory motion in the thoracic and abdominal regions of a human body is a highly discussed topic in external beam radiotherapy treatment. Errors in target/normal tissue delineation and dose calculation and the increment of the healthy tissues being exposed to high radiation doses are some of the unsolicited problems caused due to inaccurate tracking of the respiratory motion. Many related works have been introduced for respiratory motion modeling, but a majority of them highly depend on radiography/fluoroscopy imaging, wearable markers or surgical node implanting techniques. We, in this article, propose a new respiratory motion tracking approach by exploiting the advantages of an RGB-D camera. First, we create a patient-specific respiratory motion model using principal component analysis (PCA) removing the spatial and temporal noise of the input depth data. Then, this model is utilized for real-time external respiratory motion measurement with high accuracy. Additionally, we introduce a marker-based depth frame registration technique to limit the measuring area into an anatomically consistent region that helps to handle the patient movements during the treatment. We achieved a 0.97 correlation comparing to a spirometer and 0.53 mm average error considering a laser line scanning result as the ground truth. As future work, we will use this accurate measurement of external respiratory motion to generate a correlated motion model that describes the movements of internal tumors.

## 1. Introduction

Radiotherapy is one of the highly-discussed topics in the modern medical field. It has been widely used in cancer treatments to remove tumors without causing any damages to the neighboring healthy tissues. However, inaccurate system setups, anatomical motion and deformation and tissue delineation errors lead to inconsistencies in radiotherapy approaches. Respiratory-based anatomical motion and deformation largely cause errors in both radiotherapy planning and delivery processes in thoracic and abdominal regions [1,2]. With respiration, tumors in abdominal and thoracic regions can move as much as 35 mm [3,4,5,6]. As a consequence, inaccurate respiratory motion estimations directly effect tissue delineation errors, dose miss-calculations, exposure of healthy tissues to high doses and erroneous dose coverage for the clinical target volume [7,8,9,10,11].

Motion encompassing, respiratory gating, breath holding and forced shallow berating with abdominal compression are some of the existing conventional respiratory motion estimation methods [1]. Difficulties in handling patient movements, longer treatment time, patient training and discomfort are some of the most common drawbacks of these methods. On the other hand, real-time tumor tracking techniques have started to gain much attention due to their ability in actively estimating respiratory motion and continuous synchronization of the beam with the motion of the tumor.

Apart from radiotherapy, measurement of the respiration is an important task in pulmonary function testing, which is crucial for early detection of potentially fatal illnesses. Spirometer and pneumotachography are two of the well-known methods of pulmonary function testing. These methods need a direct contact with the patient while measuring and may interfere with the natural respiration. Furthermore, they measure only the full respiratory volume and cannot assess the regional pulmonary function in different chest wall behaviors. Hence, there is a need for a non-contact respiratory measurement technique, which can evaluate not only the complete, but also regional respiration.

In this paper, we investigate the feasibility of using a commercial RGB-D camera as a non-contact, non-invasive and whole-field respiratory motion-measuring device, which will enhance the patient comfort. These low-cost RGB-D cameras can provide real-time depth information of a target surface. We can use this depth information for respiratory motion measurement, but cannot achieve higher accuracy due to a considerable amount of noise in the raw depth data. Therefore, we proposed a technique of making an accurate respiratory motion model using principal component analysis (PCA) and then using that model for real-time respiratory motion measurement. First, we apply hole-filling and bilateral filtering to the first 100 raw depth frames and use that filtered depth data to create a PCA-based motion model. In the real-time respiratory motion-measuring stage, we project each depth frame to the motion model (principal components) and reconstruct back, removing the spatial and temporal noise and holes in the depth data. We can achieve higher motion measurement accuracy by using these reconstructed depth data, instead of raw depth data. The initial result of our proposed method is published in [12].

The results of this study—accurate measurements of external surface motion—can be used to predict the internal tumor motion, which is an important task of radiotherapy systems. Correspondence models that make a relationship between respiratory surrogate signals, such as spirometry or external surface motion, and internal tumor/organ motion have been studied in the literature [13,14,15,16]. Neural networks, principal component analysis and b-spline are a few example models that have been used for predicting the internal motion.

This paper is organized as follows. First, a comprehensive review of related works is presented in Section 2. An overview of the proposed method that describes the key steps and how to handle the problems existing in related works is given in Section 3. A detailed description of all of the materials and methods followed in the proposed method is presented in Section 4. The results of the experiments we conducted to evaluate the accuracy of the proposed method are given in Section 5. Finally, Section 6 concludes the paper by discussing the results and issues of the proposed method.

## 2. Related Work

The Synchrony respiratory tracking system, a subsystem of CyberKnife, is the first technology that continuously synchronizes beam delivery to the motion of the tumor [17]. The external respiratory motion is tracked using three optical fiducial markers attached to a tightly-fitting vest. Small gold markers are implanted near the target area before treatment to ensure the continuous correspondence between internal and external motion. The Calypso, the prostate motion-tracking system integrated into Varian (Varian Medical Systems, Palo Alto, CA, USA), eliminates the need for internal-external motion modeling by implanting three tiny transponders with an associated wireless tracking [18]. The BrainLAB ExacTrac positioning system uses radiopaque fiducial markers, implanted near the target isocenter, with external infrared (IR) reflecting markers [19]. Internal markers are tracked by an X-ray localization system, while an IR stereo camera tracks the external markers. The Xsight Lung Tracking system (an extension of the CyberKnife system) is a respiratory motion-tracking system of lung lesion that eliminates the need for implanted fiducial markers [20].

Another interesting respiratory motion modeling technique using 4D computed tomography (CT) images was introduced in [21], where PCA is used to reduce the motion artifacts appearing on the CT images and to synthesize the CT images in different respiratory phases. Mori et al. used cine CT images to measure the intrafractional respiratory movement of pancreatic tumors [22]. Yang et al. estimated and modeled the respiratory motion by applying an optical flow-based deformable image registration technique on 4D-CT images that were acquired in cine mode [23]. In contrast to CT, magnetic resonance imaging (MRI) provides lesser ionization and excellent soft tissue contrast that helps to achieve better characterization. Therefore, 4D and cine-MRI images have been widely used for measuring organ/tumor motion due to respiration [24,25,26,27,28]. Apart from that, researchers have been experimenting with ultrasound images for tracking organs that move with respiration [29,30].

Radiography and fluoroscopy imaging techniques such as X-ray, CT and MRI have the problems of higher cost, slow acquisition, low resolution, lower signal-to-noise ratio and especially exposure to an extra dose of radiation [2,21,31,32]. Additionally, some of these systems have the disadvantage of invasive fiducial marker implantation procedures that increase the patient preparation time and treatment time.

To avoid these problems, researchers have proposed optical methods, which mainly consist of cameras, light projectors and markers. With the advantage of non-contact measurement, optical methods have no interference with the natural respiration of the patient. Ferrigno et al. proposed a method to analyze the chest wall motion by using passive markers placed on the thorax and abdomen [33]. Motion measurement is carried out by computing the 3D coordinates of these markers with the help of specially-designed multiple cameras. In [34], the authors proposed a respiratory motion-estimation method based on coded visual markers. They also utilized a stereo camera to calculate the 3D coordinates of the markers and estimated the 3D motion of the chest wall according to the movements of the markers. Yan et al. investigated the correlation between the motion of external markers and an internal tumor target [35]. They placed four infrared reflective markers on different areas of the chest wall and used a stereo infrared camera to track the motion of the markers. Alnowami et al. employed the Codamotion infrared marker-based tracking system to acquire the chest wall motion and applied probability density estimation to predict the respiratory motion [36,37]. Some researchers have investigated respiratory motion evaluation by calculating curvature variance of the chest wall using a fiber optic sensor and fiber Bragg grating techniques [38,39]. Even though the marker-based methods provide higher data acquisition rates and accuracy, the marker attachment procedure is time consuming and results in inconveniences for the patient. Furthermore, a large number of markers is needed to achieve higher spatial resolution.

In contrast to marker-based methods, structured light techniques provide whole-field measurement with high spatial resolution. Structured light systems consist of a projector and camera and emit a light pattern onto the target surface, creating artificial correspondences. The 3D information of the target surface can be found by solving the correspondences on the captured image of the illuminated scene. Aoki et al. proposed a respiratory monitoring system using a near-infrared multiple slit-light projection [40]. Even though they were able to achieve a high correlated respiratory motion pattern to a spirometer, they could not measure the exact respiratory volume or motion due to the variable projection coverage on the chest wall, which is caused by patient movements. Chen et al. solved this problem by introducing active light markers to define the measuring boundary, offering a consistent region for volume evaluation [41]. They also used a projector to illuminate the chest wall with a structured light pattern of color stripes and a camera to capture the height-modulated images. Then, the 3D surface calculated by triangulation is used to derive the respiratory volume information. However, the long baseline and the restriction of the camera plane to be parallel to the reference frame limit the portability of this method. In [31], the authors adopted a depth sensor, which uses a near-UV structured light pattern, along with a state-of-the-art non-rigid registration algorithm to identified the 3D deformation of the chest wall and hence the tumor motion. Time of flight (ToF) is another well-known optical method that has been used by researchers for respiratory motion handling during radiotherapy [42,43,44].

With the recent advances in commercial RGB-D sensors such as the Microsoft Kinect and ASUS Xtion Pro, these have been used in a broad area of research work. Have a relatively low cost and the fact that these sensors can measure the motion without any markers or wearable devices encourage researchers to use them in respiratory motion analysis. However, the low depth resolution of these sensors, which is about 1 cm at a 2 m distance, restricts the usage mostly for evaluating respiratory functions such as respiratory rate [45,46,47,48,49,50,51], where highly accurate motion information is not needed. In the case of radiotherapy, respiratory motion induces tumor movements up to 2 cm in abdominal or thoracic regions and needs less than 1 mm accuracy in motion measurements [52]. Xia and Siochi overcome the low depth resolution of the Kinect sensor by using a translation surface, which magnifies the respiratory motion and reduces the noise of irregular surfaces [53]. A few other researchers utilized RGB-D sensors to acquire 3D surface data of the chest wall and applied PCA to capture 1D respiration curves of disjoint anatomical regions (thorax and abdomen), which is related to the principal axes [32,54]. However, the respiratory motion measurement accuracy of these methods is affected by the patient movements, as they have not provided a proper method for handling these.

## 3. Overview of the Proposed Method

In this study, we introduce a non-contact, non-invasive and real-time respiratory motion measurement technique using an RGB-D camera, which is small in size and more flexible for handling. Furthermore, we introduce a patient movement-handling method using four dot markers. These four markers define the measurement boundaries of the moving chest wall, providing a consistent region for respiratory motion estimation.

Using the RGB-D camera, we capture continuous depth images of the patient’s chest wall at 6.7 fps covering the whole thoracic and abdominal area. Then, we create a respiratory motion model by applying PCA to the first 100 frames, decomposing the data into a set of motion bases that corresponded to principal components (PCs). Before applying PCA, we use an edge-preserving bilateral filter and a hole-filling method to remove the noise and the holes of the first 100 frames.

**Figure 1:**
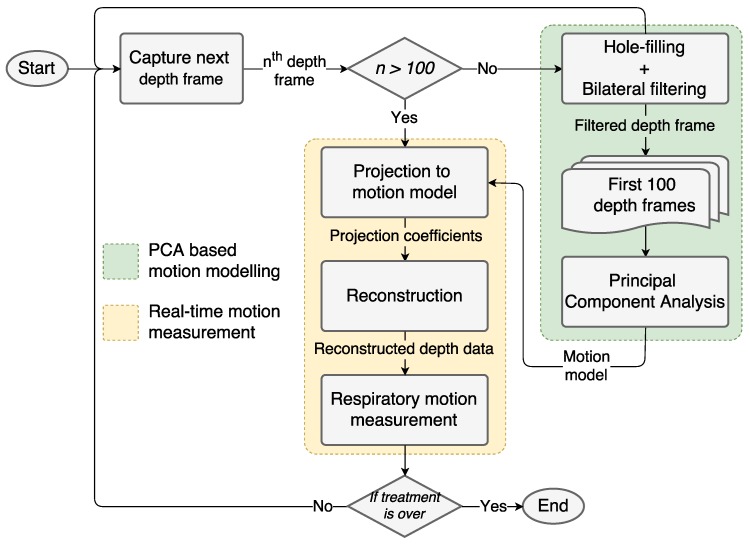
Flowchart of the proposed PCA-based respiratory motion-analyzing system. The first 100 depth frames are used to generate a PCA-based respiratory motion model. Then, that model (principal components) is used for real-time respiratory motion measurement starting from the 101st frame.

According to the experimental analysis, we found out that a respiratory motion model can be accurately obtained using the first three principal components. The remaining principal components represent the noise and motion artifact existing in the input data. We start the real-time respiratory motion measurement from the 101st frame, projecting each new depth frame onto the motion model to obtain the low-dimensional representation of the data. To evaluate the motion in metric space, depth images are reconstructed using the projection coefficient. Figure 1 shows the flowchart of the proposed respiratory motion measurement process.

Using an RGB-D camera for respiratory motion measurement has many advantages. First, compared to the CT/MRI techniques, the proposed method prevents patients from being exposed to an extra dose of radiation. The RGB-D camera is a non-contact optical method and has no interference with the natural breathing of the target. Moreover, this can give real-time depth information of the target surface. Therefore, we can provide a comfortable and efficient, but lesser duration, treatment to the patients. Compared to marker-based methods, the RGB-D camera has high spatial resolution and provides depth information of the entire target surface; hence, we can measure not only the entire chest wall motion, but also the regional motions. The RGB-D camera we use in our system provides depth data in 640×480 resolution, and we select a 200×350 ROI providing 70,000 data points for motion measurement, which is much higher than marker-based methods (as an example, [36] used a 4×4 marker grid providing only 16 data points). The smaller size and lower price of the RGB-D cameras facilitate building a more portable and inexpensive respiratory motion measurement system compared to some other optical methods.

However, there is a known problem of low accuracy of the RGB-D cameras. Depth data acquired from low-cost RGB cameras has much noise and many holes that affect the accuracy of motion measurement. Alnowami et al. and Tahavori et al. used depth data acquired from an RGB-D camera for respiratory motion measurement, but could not achieve sub-millimeter level accuracy when it comes to experiments with real persons [55,56]. Using the PCA-based motion model, we increase the motion measurement accuracy by removing the spatial and temporal noise along with the holes in the depth data. When the filtered depth data are used as the input of the PCA-based motion model, we do not need to apply bilateral filtering or hole-filling for each depth frame during real-time motion measurement. Comparing with a laser line scanner, we prove that our method can achieve sub-millimeter accuracy in respiratory motion measurement using a low-cost RGB-D camera.

## 4. Materials and Methods

### 4.1. Data Acquisition

We use an Asus Xtion PRO RGB-D camera (consisting of an RGB camera, an infrared camera and a Class 1 laser projector that is safe under all conditions of normal use) to acquire real-time depth data and RGB images of the entire thoracic and abdominal region of the target subjects. The RGB-D camera provides both depth and RGB-D images in 640×480 resolution and 30 frames per second. However, due to the process of saving data to disk for later analysis, we could acquire only about 6.7 frames per second. The OpenNI library is used to grab the depth and RGB data from the camera and to convert them to matrix format for later usage. The depth camera covers not only the intended measuring area, but also the background regions. Moreover, the coverage of the chest wall is variable due to the surface motion and the patient movements. However, we should have an anatomically-consistent measuring area during the whole treatment time for delivering the radiation dose accurately.

To handle this problem, we attach four dot markers to define a measuring boundary on the chest wall covering the whole thoracic and abdominal area. Instead of using active LED markers or retroreflective markers, which can interfere with the RGB-D camera, we use small white color circles made of sticker paper.

**Figure 2:**
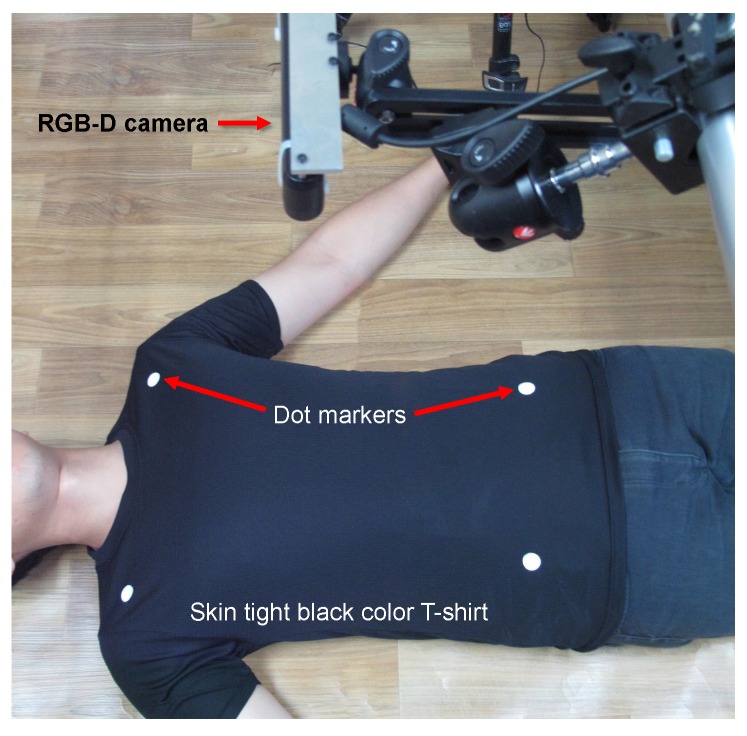
Experimental setup where the patient is laying down in the supine position wearing a skin-tight t-shirt with four white color dot markers. The RGB-D camera is placed nearly 85 cm above the patient.

After obtaining informed consent from all subjects following the institutional ethics, we collected respiratory motion data from ten healthy volunteers. All of the volunteers were advised to wear a skin-tight black color t-shirt and lay down in a supine position. The four markers are attached to the t-shirt, and the RGB-D camera is placed nearly 85 cm above the volunteer as shown in Figure 2. According to the specification of the RGB-D camera, it can provide depth information within an 80 cm to 350 cm range. However, [55] showed that the RGB-D camera gives the best accuracy within the 85 cm to 115 cm range. By keeping the camera closer to the volunteer, we can cover the measuring area with a higher number of pixels, which eventually provides more data points for motion analysis. Analyzing all of these facts, we place the RGB-D camera 85 cm above the patient. Along with the continuous depth frames, visual images are also captured using the built-in RGB camera nearly for a duration of one minute. The RGB images are used only for the purpose of detecting the markers to determine the measuring ROI.

### 4.2. Measuring Region

**Figure 3:**
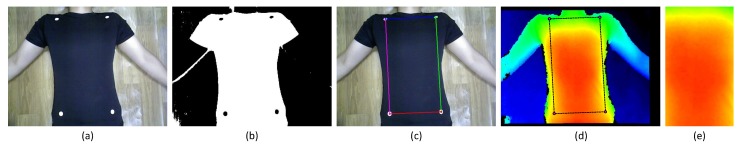
The process of rectangular ROI generation. (**a**) Captured visual image; (**b**) after binarization using Otsu’s method; (**c**) defining the measuring area after finding the center coordinates of the four markers; (**d**) identified measuring area projected onto the aligned depth image; (**e**) generated rectangular ROI using perspective transformation.

To define the measuring region, we detect the dot markers on the RGB image by applying few image processing techniques. Otsu’s global binary thresholding method followed by contour detection and ellipse fitting [57] are applied to identify the center coordinates of each dot marker accurately. Using the intrinsic and extrinsic parameters of the depth and RGB cameras, which are acquired by a calibration process [58,59], depth images are precisely aligned (with sub-pixel accuracy) to the visual (RGB) images. Therefore, the marker coordinates found on visual images can be directly used on depth images to define the ROI, which marks the measuring area. The position, shape and size of the ROI are not consistent throughout all of the depth frames due to the motion of the chest wall and the movement of the patient. In order to make it consistent, the selected ROI on every depth frame is mapped into a predefined size of a rectangular shape using projective transformation [60]. Figure 3 shows the steps followed for detecting the dot markers and creating the rectangular ROI. We use this rectangular ROI for further processing of our proposed method.

### 4.3. Respiratory Motion Modeling Using PCA

#### 4.3.1. Depth Data Pre-Processing

**Figure 4:**
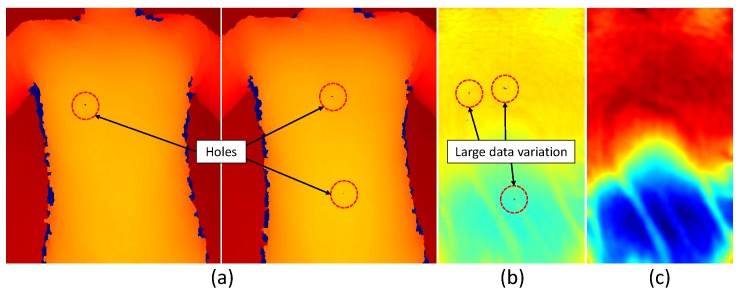
(**a**) Two example depth frames where holes appear in the chest wall region; (**b**) erroneous PCA result (eigenvector) where large data variations appear near the hole regions; (**c**) PCA result after applying hole-filling and bilateral filtering to input depth data.

We use the first 100 depth frames to create a respiratory motion model using PCA. Since we use this model for real-time respiratory motion measurement, a precise model should be created using accurate input data. Due to the slight reflection of the t-shirt and device errors, holes can appear in the same spot of the chest wall area for a few continuous depth frames as depicted in Figure 4a. Moreover, there is much noise existing in the raw depth data provided by the sensor. If we directly use these data as the input for PCA without any pre-processing, we will encounter erroneous results as in Figure 4b, where most of the data variation is concentrated in the areas of holes.

To avoid this problem, we first apply a hole-filling technique on depth images using the zero-elimination mode filter. If there are enough non-zero neighbors, this filter replaces pixels with zero depth values with the statistical mode of its non-zero neighbors. Next, we remove noise from depth images using an edge-preserving bilateral filter [61]. Figure 4c shows the PCA result when we use filtered depth data as the input.

#### 4.3.2. Principal Component Analysis

After applying filtering to the first 100 depth frames, PCA [62] is applied to make a respiratory motion model that is integrated into the major principal components. By column-wise vectorization of the depth data ($d_{i}$) on the selected rectangular ROI, we create an input data matrix *D* of dimension m×n:

(1) $$D_{m\times n}=\left[\begin{array}{c} \vec{d_{1}},\vec{d_{2}},\cdots ,\vec{d_{n}} \end{array}\right],$$

where *n* is the total number of depth frames (n=100) and *m* is number of pixels in the rectangular ROI. First, we subtract the mean vector $\vec{\bar{d}}$ calculated as:

(2) $$\vec{\bar{d}}=\frac{1}{n}\sum_{i=1}^{n}\vec{d_{i}}$$

from the input data matrix to create a normalized matrix $\hat{D}$:

(3) $$\hat{D}=\left[\begin{array}{c} \vec{d_{1}}-\vec{\bar{d}},\vec{d_{2}}-\vec{\bar{d}},\cdots ,\vec{d_{n}}-\vec{\bar{d}} \end{array}\right].$$

Since m≫n, we use Equation (4) to calculate the n×n covariance matrix *C*, reducing the dimensionality of the input data.

(4) $$C=\frac{1}{n-1}{\hat{D}}^{T}\hat{D}$$

The transformation, which maps the high-dimensional input depth data into a low-dimensional PC subspace, is obtained by solving the eigenvalues ($\lambda_{j}$) and eigenvectors ($\vec{\phi_{j}}$) of the covariance matrix using Equation (5).

(5) $$C\vec{\phi_{j}}=\lambda_{j}\vec{\phi_{j}}$$

All of the eigenvectors, which correspond to principal components, are then arranged in descending order $\left\{\vec{\phi_{1}},\vec{\phi_{2}},\vec{\phi_{3}},\cdots ,\vec{\phi_{n}}\right\}$ according to the magnitude of the eigenvalues ($\lambda_{1}\geq \lambda_{2}\geq \lambda_{3}\geq \cdots \geq \lambda_{n}$).

**Figure 5:**
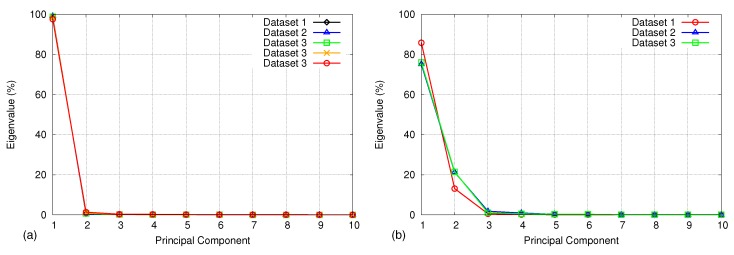
(**a**) Comparison of the first ten principal components using five sets of input data taken during regular breathing and (**b**) three sets of input data taken during irregular breathing. The first principal component is dominant over others and represents over 98% of data variance for regular breathing, while three principal components are needed to cover 98% of data variance for irregular breathing.

**Figure 6:**
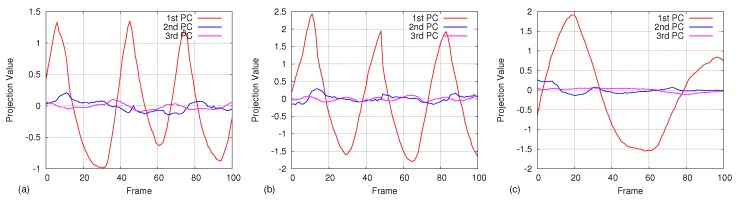
Projection results of 100 depth frames onto the first three PCs. Only the first PC shows a clear respiratory motion pattern for three datasets (**a**,**b**,**c**) taken during regular breathing.

**Figure 7:**
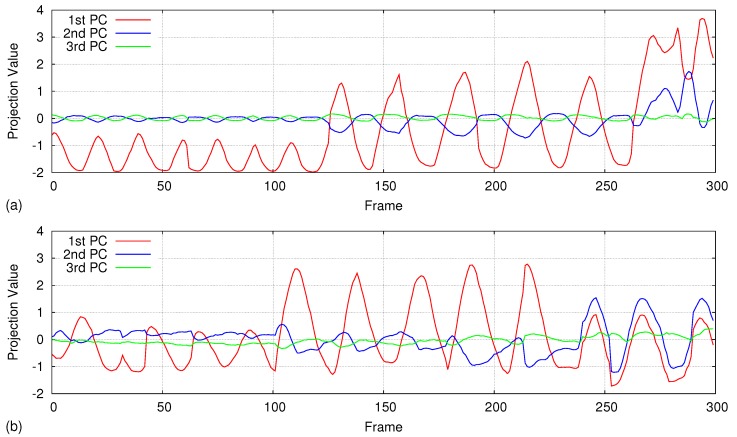
Projection results of 300 depth frames on the first three PCs for irregular breathing. The first two principal components show an apparent respiratory pattern, while the third one also shows a smaller respiratory signal. Graphs (**a**,**b**) represent two datasets.

Using an experimental analysis, we found out that the first eigenvalue dominates the rest of the eigenvalues and accounts for over 98% of the data variation during regular respiration. However, when the respiration is irregular, three eigenvalues are required to cover 98% of the data variation. Figure 5 depicts the first ten eigenvalues of the covariance matrix calculated from five samples on regular breathing and three samples on irregular breathing. Figure 6 shows three graphs of projection coefficients (explained in Section 4.4.1) corresponding to the first three principal components calculated for regular breathing, while Figure 7 shows examples of irregular breathing. An apparent respiratory motion pattern is visible only on the first PC for regular breathing, while the first three PCs show a respiratory pattern in irregular breathing. Following this analysis, we represent the respiratory motion model *W* using the first three principal components ($\vec{\phi_{1}},\vec{\phi_{2}},\vec{\phi_{3}}$), reducing the dimensionality of input depth data.

### 4.4. Real-Time Respiratory Motion Measurement

After creating a respiratory motion model using the first 100 depth frames, we start the real-time respiratory motion measurement from the 101st frame. The data we use for respiratory motion modeling should cover a few complete respiratory cycles in order to generalize the input data. By following this rule, we can make sure that the motion model represents all of the statuses of the respiratory cycle. After observing all of the experiment datasets, we empirically select 100 as the number of depth frames for PCA-based motion modeling.

#### 4.4.1. Projection and Reconstruction

We project each new depth frame $d_{i}$ (i>100) onto the motion model $W=\left[\begin{array}{ccc} \vec{\phi_{1}} & \vec{\phi_{2}} & \vec{\phi_{3}} \end{array}\right]$ in order to represent them using the first three principal components. The following equation is used as the projection operation, where $\vec{\beta_{i}}$ represents the projection coefficients.

(6) $$\vec{\beta_{i}}=W^{T}\left(\vec{d_{i}}-\vec{\bar{d}}\right)$$

Even though the calculated projection coefficients represent a clear respiratory motion, we cannot use these directly for measuring the motion as these coefficients are three separate values in the principal component domain instead of the metric domain. Therefore, the following equation is used to reconstruct the depth data ($\hat{\vec{d_{i}}}$), which is in the metric domain, from the projection coefficient.

(7) $$\hat{\vec{d_{i}}}\approx \vec{\bar{d}}+W\vec{\beta_{i}}$$

**Figure 8:**
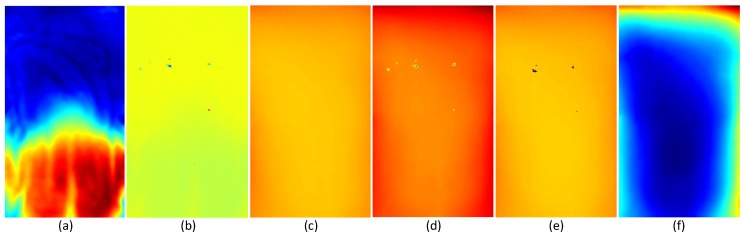
(**a**) PCA result (first eigenvector) using bilateral filtering and hole-filling; (**b**) PCA result (erroneous) without using bilateral filtering and hole-filling; (**c**) example input depth image without any holes; (**d**) reconstruction results of (**c**) using the incorrect PCA results shown in (**b**); (**e**) example input depth image with few holes; (**f**) reconstruction results of (**e**) using the PCA results shown in (**a**).

Here, the advantage is that we do not need to apply hole-filling or denoising filters to the depth data that we use for real-time respiratory motion measurement. By reconstructing the depth images using the motion model, we can remove the spatial and temporal noise, as well as the holes in the data. Figure 8 depicts the advantage of applying bilateral filtering and hole-filling to the input depth images for PCA. Figure 8a,b shows the PCA results with and without using filtering on PCA input data, respectively. As shown in Figure 8c,d, if we use the erroneous PC for projection and reconstruction, many holes and much noise will appear on the reconstructed depth data even if there are no holes in the input data. In contrast to that, if we use an accurate PC for projection and reconstruction, we can remove the holes and noise appearing in the input depth data by reconstructing it as shown in Figure 8e,f.

#### 4.4.2. Motion Measurement

**Figure 9:**
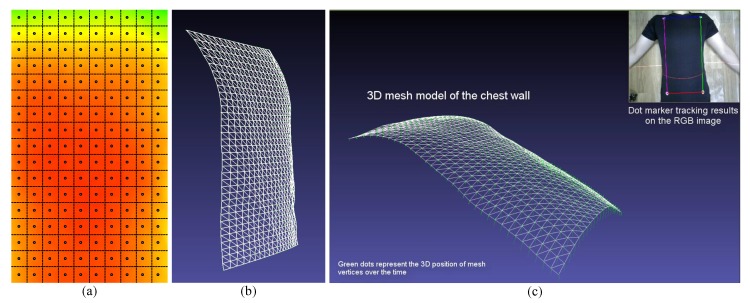
The surface mesh generation process. (**a**) The rectangular ROI of the reconstructed depth is further divided into smaller square ROIs; (**b**) a surface mesh is generated by finding the 3D coordinate of the midpoints of smaller ROIs using the average depth value of the region; (**c**) a selected frame of a video sequence, which shows the motion of the chest wall in a 3D viewer using a mesh model. Green dots represent the 3D position of mesh vertices over time.

We use these reconstructed depth data for respiratory motion measurements. The rectangular ROI of the reconstructed depth data is further divided into smaller regions as in Figure 9a to separately measure the motion in smaller regions. Average depth values of these smaller regions along with 2D image coordinates and intrinsic camera parameters are used to calculate the 3D (X, Y and Z) coordinates of the mid-points. Then, we use these 3D coordinates to construct a surface mesh model composed of small triangles as in Figure 9b,c, which can be used to represent the chest wall surface and its motion clearly. We define the initial frame (101st frame) as the reference frame and calculate the motion of the remaining frames using the depth difference between the current frame and the reference frame.

### 4.5. Evaluation of the Accuracy

**Figure 10:**
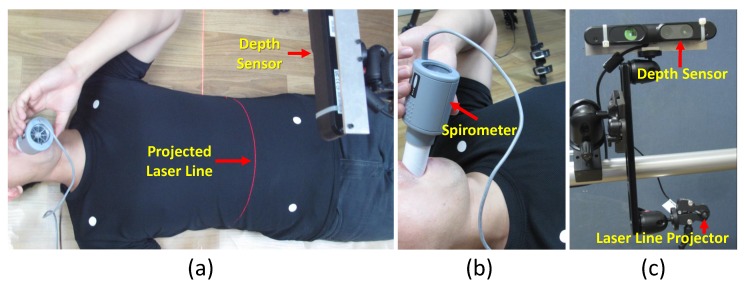
Experimental setup for evaluating the accuracy of the proposed method using a spirometer and a laser line scanner. (**a**) Volunteers are advised to lay down in the supine position and breath only through the spirometer. The RGB-D camera and laser line projector are placed above the volunteer, and the laser line is projected onto the abdomen area. (**b**) CareFusion SpiroUSB™spirometer. (**c**) The configuration of the RGB-D camera and laser line projector.

We propose an experimental setup as shown in Figure 10 for evaluating the accuracy of the proposed method. First, our proposed method is compared with a spirometer, which measures the air flow volume using a mouthpiece device, and then with a laser line scanner, which provides very accurate 3D reconstruction results.

#### 4.5.1. Comparison with Spirometer

We compared the respiratory motion pattern generated using the proposed method with a spirometer, which has been used for evaluating the accuracy of RGB-D camera-based respiratory function evaluation methods [41,63,64]. During this experiment, the patient breathed through a calibrated spirometer (SpiroUSB™, CareFusion) to record the airflow volume while the depth camera captured the chest wall motion simultaneously (see Figure 10a,b). The spirometer provides the air flow volume in liters, not the respiratory motion in millimeters. Therefore, with the help of surface mesh data, we developed a method to measure the volume difference of the current frame compared to a reference frame. We found the volume difference by calculating the sum of the volume of small prisms created by the triangles in the surface mesh of the current frame and their projection on the reference plane as the top and bottom surfaces.

First, these prisms were further divided into three irregular tetrahedrons. Then, the volume of a tetrahedron was calculated using Equation (8), where $a(a_{x},a_{y},a_{z})$, $b(b_{x},b_{y},b_{z})$, $c(c_{x},c_{y},c_{z})$ and $d(d_{x},d_{y},d_{z})$ represent the 3D coordinates of the four vertices.

(8) $$V=\frac{det(A)}{6},\;A=\left[\begin{array}{cccc} a_{x} & b_{x} & c_{x} & d_{x}\\ a_{y} & b_{y} & c_{y} & d_{y}\\ a_{z} & b_{z} & c_{z} & d_{z}\\ 1 & 1 & 1 & 1 \end{array}\right]$$

#### 4.5.2. Comparison with Laser Line Scanning

Laser line scanning, which is well known for providing high accuracy (<0.1 mm) [65], is a 3D reconstruction method consisting of a laser line projector and a camera. We used this method to reconstruct a specific position of the chest wall accurately and to compare it with the PCA reconstruction results. The setup for this experiment consists of a laser line projector and the RGB-D camera as shown in Figure 10c. We projected the laser line onto the abdominal area of the target chest wall and captured the illuminated scene using the visual (RGB) camera of the RGB-D sensor. We prepared 15 datasets (D01, D02, ..., D15) from ten healthy volunteers ranging in age from 24 to 32 who participated in the data capturing process. Volunteer information is given in Table 1.

First, we calibrated the laser line projector and the RGB camera to find the 3D plane equation of the laser line with respect to the camera coordinate system using a checkerboard pattern [65,66]. Then, we separated the measuring area from the rest of the image by defining a rectangular ROI on the RGB images the same as on the depth images. We took the red channel of the RGB image, applied Gaussian smoothing and fit a parabola to each column of the ROI image according to the pixel intensities. Then, by finding the maximum of the parabola, which corresponds to the laser line location, we can identify the 2D image coordinates of it with sub-pixel level accuracy. We projected these image coordinates to the 3D laser plane using the intrinsic camera parameters and calculated the 3D coordinates by finding the ray-plane intersection points. These 3D coordinates are referred to as *laser reconstruction* in the remainder of this paper. Next, we projected the 2D coordinates of the laser line onto the reconstructed depth image $\hat{\vec{d_{i}}}$ to identify the 3D coordinates of the laser line according to the proposed PCA-based method and referred to this as *PCA reconstruction*.

The purpose of the proposed method is not to reconstruct the chest wall surface, but to measure the chest wall motion accurately. Therefore, instead of comparing the direct 3D reconstruction results, we compared the respiratory motion; defined as the depth difference between the current frame and reference frame. We chose the 101st frame as the reference frame, as it is the starting frame of real-time respiratory motion measurement. To have a quantitative comparison, we selected five points (P1,P2,⋯,P5) across the laser line and found the motion error of each point separately for 100 frames. By taking the laser line reconstruction as the ground truth, we calculated the motion error $E_{ij}$ of the *j*-th point on the laser line of *i*-th frame (1≤j≤5 and 1≤i≤100) using:

(9) $$E_{ij}=\left|\left(D_{ij}^{L}-D_{rj}^{L}\right)-\left(D_{ij}^{P}-D_{rj}^{P}\right)\right|,$$

where $D_{ij}$ is the depth value of the *j*-th point on the laser line of the *i*-th frame. *L* and *P* represent the laser reconstruction and PCA reconstruction, respectively, while *r* represents the reference frame.

## 5. Results

First, we present the accuracy evaluation results of the proposed respiratory motion measurement method compared to the spirometer and laser line scanner. With the use of the spirometer, we examined the respiratory pattern using volume changes. The laser line scanner was used to analyze the motion measurement accuracy of the proposed method. Later, we compared our method with bilateral filtering and then conducted isovolume maneuver to show the advantages of the proposed method over existing ones. Finally, we analyzed how the proposed method works in a condition of longer and irregular breathing. All of these experiments were performed in a general laboratory environment, and the software components were implemented using C++ language with the help of OpenCV and OpenNI libraries.

### 5.1. Comparison of Respiratory Pattern with Spirometer

**Figure 11:**
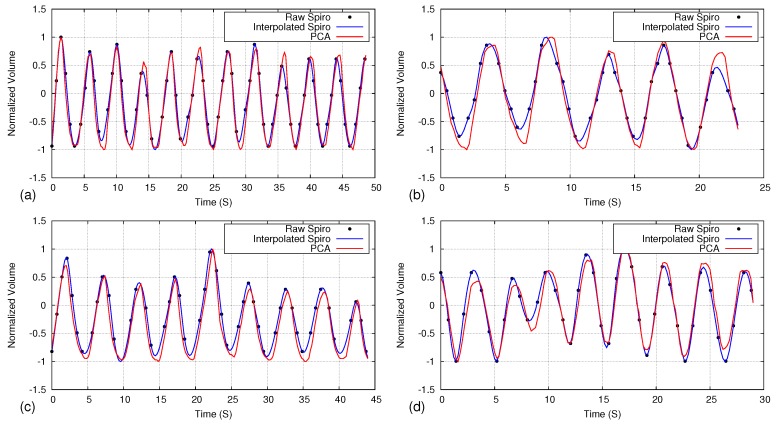
Comparison of respiratory volume measurement (normalized into −1:1 range) using the proposed method (PCA) and a spirometer. Graphs (**a**–**d**) represent the selected four different datasets. Black dots represent the original data points of the spirometer, while the blue line represents the interpolated data.

Figure 11 depicts the volume comparison graphs of the spirometer and the proposed PCA-based method. The sample rate of the spirometer is lower than the RGB-D camera. Therefore, we applied b-spline interpolation on available spirometer data to generate a smooth motion curve to achieve a similar frame interval as the RGB-D camera.

The magnitude of the respiratory volume is different between the spirometer and the proposed method, as the measuring area and methodology are different. Therefore, we compared the data by normalizing it to a −1:1 range. As shown in Figure 11, the proposed method could generate respiratory motion patterns very similar to the spirometer with a 0.97 average correlation.

### 5.2. Accuracy Analysis Using Laser Line Scanning

**Figure 12:**
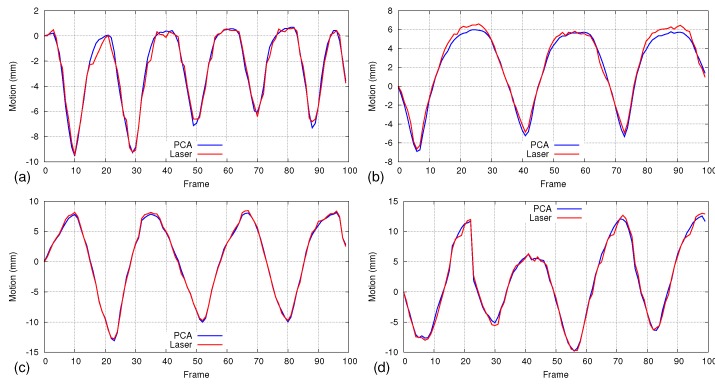
Comparison of respiratory motion measurement using the proposed method (PCA) and laser line scanning. Measurements are taken from different places on the projected laser line. The 101st frame of the dataset is selected as the reference frame, and we measure the motion of remaining frames with respect to it until the 200th frame. Graphs (**a**–**d**) show the motion measurement results of four different datasets.

**Figure 13:**
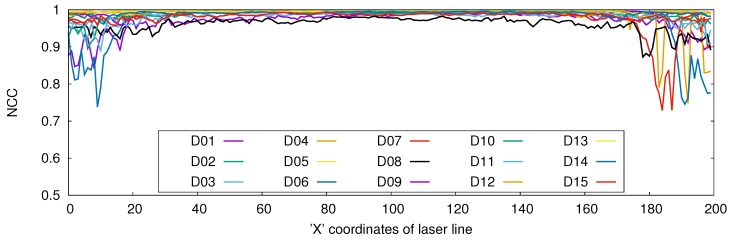
Normalized cross-correlation (NCC) between PCA and laser scanning across 100 frames. NCC is calculated for each point on the laser line along the X-axis separately.

Table 2 gives the motion error results of the five points on the laser line, calculated from 15 datasets. We summarized the data on the table as the average, maximum and standard deviation of the motion error ($E_{ij}$) over 100 frames. The average motion error of all datasets on all five points is 0.53±0.05 mm. As a qualitative comparison, motion graphs of four datasets calculated on four different points of the laser line are depicted in Figure 12. As a further analysis, we calculated the normalized cross-correlation (NCC) between the PCA motion ($D_{ix}^{P}-D_{rx}^{P}$) and laser line motion ($D_{ix}^{L}-D_{rx}^{L}$) for each *x* coordinate of the laser line over 100 frames. The graph in Figure 13 shows the NCC results, which was separately calculated for each X-coordinate of the laser line for all 15 datasets. The results indicate a very high correlation between the two motion estimation methods as the average NCC for all of the datasets is 0.98±0.0009.

### 5.3. Comparison with Bilateral Filtering

**Figure 14:**
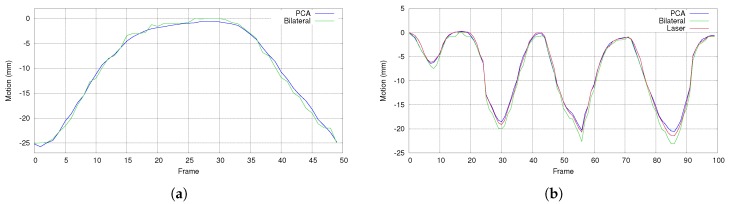
Comparison of the proposed PCA-based method and bilateral filtering. (**a**) A part of the motion comparison graph. The proposed PCA-based method provides a smooth curve, while bilateral filtering gives a rough curve with more temporal noise. (**b**) Comparison of the proposed PCA-based method and bilateral filtering with laser line scanning.

To show the advantages, we compared our proposed method with bilateral filtering. In our method, hole-filling and bilateral filtering are applied only to the first 100 frames that we used as the input for PCA, and we do not use this during real-time respiratory motion measurements. During this experiment, we measured the respiratory motion by applying bilateral filtering and hole-filling to all frames and without using PCA, and the results are compared with the proposed PCA-based method. Figure 14a shows a part of the motion comparison graph, where the bilateral filtering gives a rough curve with more temporal noise, while the proposed method gives a smoother curve with less temporal noise. The reason is that PCA provides both spatial and temporal filtering, not like bilateral filtering, which provides only spatial filtering.

Furthermore, Figure 14b compares the proposed method and bilateral filtering with a very accurate 3D reconstruction method of laser line scanning (details are given in Section 4.5.2). Considering the laser reconstruction as the ground truth, we calculated the motion error (Equation (9)) of the proposed method and bilateral filtering on a selected location of the chest wall. In the case of the motion comparison provided in Figure 14b, the average error is 0.35±0.06 mm for the proposed method and 0.85±0.08 mm for the bilateral filtering.

### 5.4. Isovolume Maneuver

**Figure 15:**
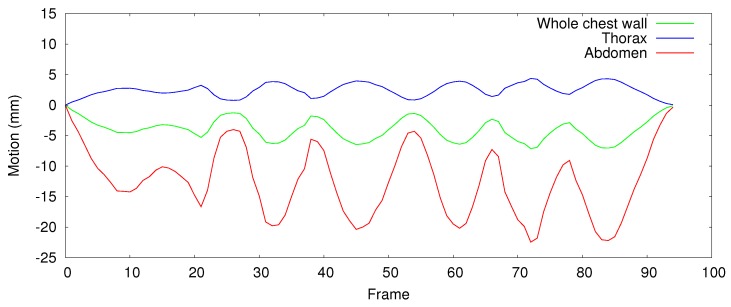
Respiratory motion graph of a volunteer performing the isovolume maneuver. The opposite phase of the whole thorax and the whole abdomen motion reflect the volume exchange between them.

We conducted an isovolume maneuver to emphasize the capability of the regional respiratory motion measurement of the proposed method. During the test, the subjects are advised to hold their breath without air flow, but exchanging the internal volume between thorax and abdomen. Then, we measured the motion of whole chest wall (which is covered by the four dot markers) and the regional motion of thorax and abdomen separately, presented in Figure 15. We used a few additional markers to separate the thorax and abdomen area on the chest wall. Theoretically, there should be no volume changes for the whole chest wall, but as we measure the depth difference in an ROI defined by the markers, which does not cover the entire chest wall area exactly, a motion pattern appears on the whole chest wall. However, opposite phases of the whole thorax and the whole abdomen motion with −0.99 cross-correlation reflecting the volume exchange between them, which we cannot determine using a respiratory volume-measuring devices such as the spirometer.

### 5.5. Handling Irregular Breathing

**Figure 16:**
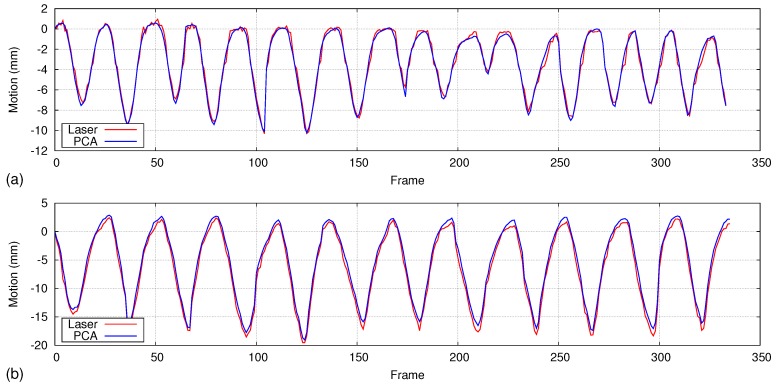
Motion comparison graphs generated for a regular respiratory patterns over a longer duration (350 frames). The first 100 frames are used for PCA, and only the first principal component is used as the motion model. All frames are then used for accuracy analysis. Higher accuracy could be achieved even though only the first PC is used for reconstruction. Graphs (**a**,**b**) show the motion comparison results of two different datasets.

We analyze how the motion model generated using the first 100 frames affects the accuracy during longer and irregular breathing. For regular respiration that does not have much variation in respiratory rate and volume, only the first principal component is enough to accurately measure the motion. Figure 16 shows two graphs of regular respiratory motion that were calculated over 350 frames compared with the laser line scanning (details are given in Section 4.5.2). Even though we use only the first principal component calculated over 100 depth frames, the average error is about 0.3 mm and 0.8 mm for the two graphs, respectively.

**Figure 17:**
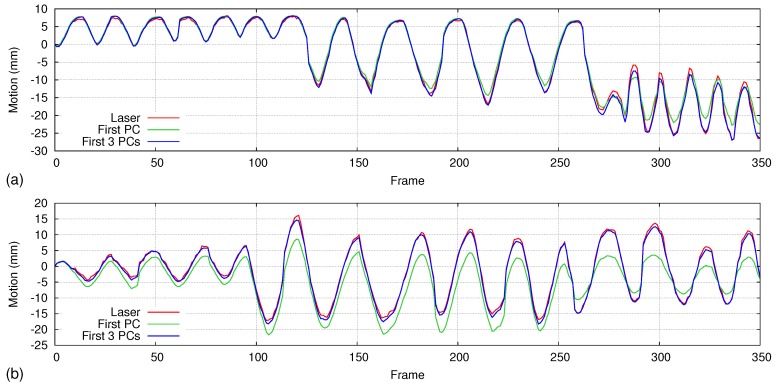
Motion comparison graphs generated for irregular respiratory patterns over a longer duration (350 frames). The first 100 frames are used for PCA, and the first principal component and first three principal components are used as the motion models, respectively. All frames are then used for accuracy analysis. A large difference appears between the laser scanner and PCA method when we are using only the first principal component. Higher accuracy could be achieved when we are using the first three principal components as the motion model. Graphs (**a**,**b**) show the motion comparison results of two different datasets.

However, during irregular breathing (respiratory rate and amplitude change time to time), accuracy gets lower when we are using only the first principal component as the motion model. As shown in Figure 17, the large difference compared to the laser line scanning proves that only the first principal component is not enough for handling irregular respiratory motions. Therefore, we redo the accuracy analysis including the first three principal components of the motion model and draw the results on the same graph. Using the first three principal components, we could achieve sub-millimeter accuracy (∼0.5 mm) even if the respiratory pattern of the first 100 frames is entirely different from rest of the data.

As a further refinement step for a very long treatment duration, we can update the motion model by recalculating the principal components with a new set of depth data at regular intervals.

## 6. Discussion and Conclusions

We have proposed a patient-specific external respiratory motion analyzing technique based on PCA. A commercial RGB-D camera was used to acquire the depth data of the target respiratory motion, and PCA was applied to find a motion model corresponding to the respiration. Four dot markers attached to the chest wall were used to define an anatomically-consistent measuring region throughout the measuring period. Using an experimental analysis, we found out that only the first three principal components are sufficient to represent the respiratory motion while the rest of the principal components represent patterns of small perturbations. Therefore, all of the depth data were projected onto the first three principal component and reconstructed removing the spatial and temporal noise existing in the input data.

For the convenience of the volunteers who participated in the laboratory-level experiments, we allowed them to wear a black-colored t-shirt and attached white color dot markers on it. Even though we use a tight-fitting t-shirt, a few wrinkles can appear within the chest wall area and affect the accuracy of the results. Therefore, we recommend not using any clothing that covers the measuring region during the clinical treatment process. We can select dot markers with an apparent color difference with the patient’s skin color and directly attach them to the patient’s body. Furthermore, it is advisable to attach the dot markers on four locations of the chest wall where there is no compelling motion due to the respiration, such as the end of the collar bones and hip bones.

During respiratory motion modeling using PCA, we used the first 100 depth frames as the input data. The criterion for selecting this number is that input depth data should cover a few complete respiratory cycles. All of our experiment datasets satisfy this criterion within 100 frames. The frame rate during the experiments was about 6.7 fps on average because it takes time for writing/reading data to hard disk frame by frame. However, during real respiratory motion measurement sessions, reading and/or writing data to a hard disk is not necessary; thus, we can achieve a frame rate of around 20 fps. The frame rate was very stable during the experiments with only a 0.4 fps standard deviation.

The accuracy of the proposed method was first evaluated using a spirometer, which has an accuracy level of 3%. Even though the magnitude of the measured volume was different, the spirometer and the proposed method were highly correlated in motion pattern (0.97 average correlation). Second, a laser line scanning technique, which is well known for high accuracy, was used to analyze the motion measurement accuracy of the proposed method. A laser line that was projected onto the abdominal area of the subject was reconstructed using a laser line scanning technique and compared with the proposed PCA reconstruction method. The motion of the projected laser line is measured using the both reconstruction results with respect to a reference frame. We could achieve high correlation (0.98 NCC) between the laser line scanner and the proposed method. Considering the laser scanning results as the ground truth, the measured average motion error of the proposed method is 0.53 mm, which is very comparable to commercial respiratory tracking systems according to Table 3.

The proposed method provides not only a high accuracy, but also a very simple system setup, which is very flexible and portable. With the advantage of non-contact measurement, the proposed method has no interference with the patient’s respiration and, hence, provides more accurate measurements. Furthermore, the proposed method has the advantage of measuring the motion in a particular location of the chest wall, instead of measuring the motion of the whole chest wall at once.

Finding a motion model that can be used to correlate the external respiratory motion with internal tumor motion has been discussed in the literature [14,15,16]. Linear, polynomial, b-spline and PCA-based models are a few techniques that have been investigated so far. As future work, we are also planning to work on finding a correlation model, that can be employed to measure internal tumor motion, by using external surface motion as the surrogate input data. Furthermore, we are planning to test the proposed system in a real clinical environment using patients with different demographic and clinical properties.

## Figures and Tables

**Table 1:** Clinical and demographic information of the volunteers who participated in the experiments.

Volunteer	Gender	Age (years)	BMI (kg/m${}^{2}$)	Datasets
1	M	29	26.4	D01, D02
2	M	32	28.7	D03
3	M	26	27.4	D04, D05
4	M	27	21.5	D06, D07
5	M	25	26.9	D08
6	M	28	26.5	D09
7	M	27	19.3	D10, D11
8	M	24	24.3	D12, D13
9	M	30	20.9	D14
10	M	25	24.0	D15

**Table 2:** Motion error of the proposed PCA-based method compared to laser line scanning calculated on five locations of the laser line for 15 datasets. All data are given in mm.

Position	Parameters	D01	D02	D03	D04	D05	D06	D07	D08	D09	D10	D11	D12	D13	D14	D15	Average
P1	Average	0.23	0.66	0.18	0.27	0.39	0.36	0.36	0.21	0.83	0.45	0.32	0.36	0.94	0.43	0.55	0.44
	Max.	0.92	2.69	0.66	1.14	0.96	1.41	1.41	0.77	1.91	1.45	1.05	1.47	1.89	1.24	1.51	1.37
	Standard deviation	0.19	0.66	0.13	0.24	0.22	0.32	0.32	0.16	0.47	0.31	0.23	0.29	0.50	0.27	0.38	0.31
P2	Average	0.39	0.34	0.33	0.52	0.22	1.09	0.47	0.47	0.85	0.46	0.30	0.50	0.52	0.97	0.38	0.52
	Max.	1.10	1.34	0.84	1.62	0.66	1.87	1.37	1.31	1.72	1.34	0.79	1.55	1.56	2.51	1.38	1.40
	Standard deviation	0.25	0.31	0.21	0.38	0.16	0.40	0.30	0.33	0.46	0.32	0.19	0.34	0.40	0.66	0.29	0.33
P3	Average	0.31	0.85	0.42	0.50	0.59	0.41	0.44	0.74	0.78	0.70	0.40	0.63	1.04	0.57	0.64	0.60
	Max.	1.09	1.90	1.18	1.29	1.39	1.28	1.59	1.81	1.97	1.83	1.03	1.89	2.55	1.56	1.82	1.61
	Standard deviation	0.25	0.44	0.26	0.32	0.34	0.31	0.34	0.44	0.50	0.46	0.26	0.47	0.65	0.36	0.40	0.39
P4	Average	0.42	0.27	0.28	0.51	0.34	0.40	0.36	0.38	1.18	1.55	0.69	0.50	0.74	0.49	0.41	0.57
	Max.	0.95	1.38	0.77	1.72	0.91	0.90	1.03	1.24	2.45	3.18	1.52	1.52	1.86	1.11	1.02	1.44
	Standard deviation	0.21	0.27	0.19	0.46	0.23	0.24	0.26	0.27	0.71	0.67	0.32	0.43	0.43	0.32	0.24	0.35
P5	Average	0.32	0.43	0.33	0.29	0.51	0.89	0.53	0.70	0.37	0.43	0.38	0.70	0.87	0.63	0.73	0.54
	Max.	0.89	2.23	0.96	1.61	0.97	1.68	1.46	1.59	1.27	1.35	1.02	2.04	2.16	1.63	1.79	1.51
	Standard deviation	0.22	0.49	0.21	0.27	0.23	0.37	0.40	0.40	0.29	0.33	0.25	0.59	0.56	0.37	0.42	0.36

**Table 3:** Accuracy comparison of the proposed method with related respiratory motion tracking methods.

System	Accuracy
Synchrony [17]	<1.5 mm
ExacTrac [19,67]	<1.0 mm
Calypso [18]	<1.5 mm
Yang et al. [23]	1.1±0.8 mm
Chen et al. [41]	4.25±3.49%
Alnowami et al. [55]	3.1±0.6 mm
Proposed Method	0.53±0.25 mm

## Supplementary Materials

The Supplementary Materials are available online at http://www.mdpi.com/1424-8220/17/8/1840/s1.
